# Measurement of IFN-γ and IL-2 for the assessment of the cellular immunity against SARS-CoV-2

**DOI:** 10.1038/s41598-024-51505-w

**Published:** 2024-01-11

**Authors:** Guillem Safont, Raquel Villar-Hernández, Daria Smalchuk, Zoran Stojanovic, Alicia Marín, Alicia Lacoma, Cristina Pérez-Cano, Anabel López-Martínez, Bárbara Molina-Moya, Alan Jhunior Solis, Fernando Arméstar, Joan Matllo, Sergio Díaz-Fernández, Iris Romero, Irma Casas, Kevin Strecker, Rosemarie Preyer, Antoni Rosell, Irene Latorre, Jose Domínguez

**Affiliations:** 1 Institut d’Investigació Germans Trias i Pujol, Barcelona, Spain; 2 CIBER de Enfermedades Respiratorias, Instituto de Salud Carlos III, Madrid, Spain; 3 Departament de Genètica i Microbiologia, Universitat Autònoma de Barcelona, Barcelona, Spain; 4 Genome Identification Diagnostics GmbH (GenID), Straßberg, Germany; 5 Odesa I. I. Mechnykov National University, Odesa, Ukraine; 6 Pulmonology Department, Hospital Universitari Germans Trias i Pujol, Badalona, Spain; 7 Basic Unit for the Prevention of Occupational Risks (UBP), Hospital Universitari Germans Trias i Pujol, Badalona, Spain; 8 Intensive Care Medicine Department, Hospital Universitari Germans Trias I Pujol, Badalona, Spain; 9 Preventive Medicine Department, Hospital Universitari Germans Trias i Pujol, Badalona, Spain

**Keywords:** Immunology, Microbiology, Biomarkers, Diseases

## Abstract

The study of specific T-cell responses against SARS-CoV-2 is important for understanding long-term immunity and infection management. The aim of this study was to assess the dual IFN-γ and IL-2 detection, using a SARS-CoV-2 specific fluorescence ELISPOT, in patients undergoing acute disease, during convalescence, and after vaccination. We also evaluated humoral response and compared with T-cells with the aim of correlating both types of responses, and increase the number of specific response detection. Blood samples were drawn from acute COVID-19 patients and convalescent individuals classified according to disease severity; and from unvaccinated and vaccinated uninfected individuals. IgGs against Spike and nucleocapsid, IgMs against nucleocapsid, and neutralizing antibodies were also analyzed. Our results show that IFN-γ in combination with IL-2 increases response detection in acute and convalescent individuals (p = 0.023). In addition, IFN-γ detection can be a useful biomarker for monitoring severe acute patients, as our results indicate that those individuals with a poor outcome have lower levels of this cytokine. In some cases, the lack of cellular immunity is compensated by antibodies, confirming the role of both types of immune responses in infection, and confirming that their dual detection can increase the number of specific response detections. In summary, IFN-γ/IL-2 dual detection is promising for characterizing and assessing the immunization status, and helping in the patient management.

## Introduction

The coronavirus disease 2019 (COVID-19) pandemic caused by the severe acute respiratory syndrome coronavirus-2 (SARS-CoV-2) has been the source of a great number of infections, hospitalizations, and deaths worldwide^1^. Although a lot of efforts to control the pandemic, such as developing vaccines and achieving herd immunity, have been accomplished, the post-acute phase of the pandemic is still a concern, as host immunity differs among individuals leading to different disease outcomes^2,3^. A well-stablished specific adaptive response is necessary to eliminate the virus and avoid an aggravation of the disease; however, an imbalanced immune response can lead to a worse outcome due to lymphopenia or inflammation’s exacerbation^4,5^. In addition, this response is elemental in the protection against severe outcomes and reinfections^6,7^. Therefore, the study and measurement of T-cell and humoral responses against SARS-CoV-2 are relevant for understanding the correlates of protection to reinfection and for the proper clinical management of COVID-19.

The study of IFN-γ release by T-cells has been widely used and measured by IFN-γ release assays (IGRAs) to evaluate adaptive cellular responses, as is the case for *Mycobacterium tuberculosis* infection diagnosis^8,9^. IFN-γ secretion is involved in multiple functions, including increasing antigen presentation, inducing antiviral status (prevention of viral replication and induction of apoptosis) and stimulating the expression of numerous genes related to the inflammatory process^10^. Recently, the utility of measuring the specific IFN-γ released by T-cells in the context of SARS-CoV-2 has also been assessed, showing that IFN-γ is increased during convalescence compared to the acute disease phase. In addition, a large proportion of fully vaccinated individuals (with two doses of a mRNA SARS-CoV-2 vaccine) do not show IFN-γ release^11,12^. Although a good correlation between humoral and IFN-γ T-cell responses has been previously reported^13,14^ a higher presence of humoral response is usually found in vaccinated and convalescents^11^. These findings show that IFN-γ T-cell responses can give a great overview of the specific SARS-CoV-2 cellular immunity, and that the assessment of other cytokines can provide more information on individuals’ immunological status and outcome, especially in cases with a lack of IFN-γ response^15^. IL-2-secreting T-cells have been proven to be essential in the modulation of the development, homeostasis, and regulation of T-cells, having a very important role in the adaptive immune response. IL-2 secretion is crucial for memory T-cell development, and their proliferation and maintenance when facing a specific antigen^16–18^. Therefore, analyzing the production of this cytokine against specific SARS-CoV-2 antigens can contribute to having a better picture of memory T-cell responses and protection after vaccination and throughout convalescence^15,19,20^.

Despite the many efforts to understand T-cell responses against SARS-CoV-2, more studies are required to shell the complexity of these responses during infection and to characterize the long-term immunity conferred by infection and vaccination. As IFN-γ and IL-2 are cytokines with a key role in the adaptive immune response during acute infection and T-cell memory, we hypothesize that the combined study of both cytokines will help in the management of SARS-CoV-2 infection, providing information on the immunological status in different clinical situations. Therefore, the main objective of this study was to assess the dual IFN-γ and IL-2 detection, using a SARS-CoV-2 specific fluorescence ELISPOT, in patients undergoing acute disease, during convalescence, and after vaccination. Additionally, as previously seen in other studies, humoral and T-cell response is impaired in some situations, thus, in this study, IgG, IgM, and neutralizing antibodies were also evaluated and compared with T-cells with the aim of correlating both types of responses, and increase the number of specific response detection.

## Results

### IFN-γ and IL-2 T-cell responses increased the number of positive responses

A total of 263 samples from 232 individuals were included in the study from July 2020 to May 2022. Ninety-three (35.4%) were from uninfected participants, 66 (25.1%) were from 35 acute patients, and 104 (39.5%) were from individuals during the convalescent phase. Overall, 19 of the 263 samples (7.2%) were indeterminate for CoV-iSpot and therefore excluded from the analysis. From the indeterminate samples, 11 were from acute COVID-19 patients, 7 from convalescent, and 1 from an uninfected unvaccinated participant. Ten samples (3.8%) were indeterminate only for IFN-γ and 15 (5.7%) for IL-2; 6 samples were indeterminate for both tests.

The number of individuals detected with immune response against SARS-CoV-2 increased when IFN-γ and also IL-2 T-cell response was studied [76.6% (187/244)]. The pancoronavirus panel triggered an IFN-γ or IL-2 T-cell positive responses in 35.2% and 28.7% of the samples, respectively (Supplementary Table 1). The pancoronavirus panel is a pool of spike, nucleocapsid, membrane, envelope and orf1 protein (conservative region) of the coronavirus family. According with the manufacturer description, the pool excludes homologies with the SARS-CoV-2 panel. Therefore, having a positive response for this particular panel means a response or previous exposition to seasonal coronaviruses. Positive responses for one of the cytokines and negative for the other were also analyzed for the SARS-CoV-2 specific panel (Supplementary Table 2).

### IFN-γ and IL-2 T-cell responses differ according to the clinical situation

T-cell responses in unvaccinated uninfected individuals were only found in 4 out of 20 individuals (20%), 3 (15%), and 1 (5%) of them being positive for IFN-γ and IL-2 respectively. These results evidence a previous SARS-CoV-2 infection not detected before the inclusion. Vaccinated uninfected individuals showed a lower rate of positive IFN-γ T-cell response than IL-2 [68% (49/72) for IFN-γ response vs 81% (58/72) for IL-2]. Sixty of the 72 vaccinated individuals (83%) showed a positive response for either IFN-γ or IL-2 responses (Table 1).

**Table 1:** Percentage of samples with positive T-cell responses against SARS-CoV-2 peptides (%).

	IFN-γ^+^	IL-2^+^	IFN-γ^+^ and/or IL-2^+^
Uninfected (n = 92)	52/92 (56.5)	59/92 (64.1)	64/92 (70)
Unvaccinated (n = 20)	3/20 (15)	1/20 (5)	4/20 (20)
Vaccinated (n = 72)	49/72 (68)	58/72 (81)	60/72 (83)
Acute disease (n = 55)	34/55 (61.9)	31/55 (56.4)	36/55 (65.4)
Mild (n = 5)	2/5 (40)	1/5 (20)	2/5 (40)
Moderate (n = 4)	2/4 (50)	2/4 (50)	3/4 (75)
Severe NIV (n = 31)	22/31 (71)	22/31 (71)	23/31 (74.2)
Severe IMV (n = 8)	6/8 (75)	5/8 (62.5)	6/8 (75)
Dead (n = 7)	2/7 (28.6)	1/7 (14.3)	2/7 (28.6)
Convalescent (n = 97)	80/97 (82.5)	84/97 (86.6)	87/97 (89.7)
Mild (n = 23)^a^	15/23 (65.2)	16/23 (69.6)	17/23 (73.9)
Moderate (n = 19)^a^	17/19 (89.5)	16/19 (84.2)	18/19 (94.7)
Severe NIV (n = 26)^a^	21/26 (80.8)	23/26 (88.5)	23/26 (88.5)
Severe IMV (n = 29)^a^	27/29 (93.1)	29/29 (100)	29/29 (100)

Positivity percentages were calculated excluding the indeterminate results from the total number of samples tested for each group.

*NIV* non-invasive ventilation, *IMV* invasive mechanical ventilation.

^a^Severity considered during their acute COVID-19 episode.

Acute COVID-19 patients had a slightly higher rate of positive IFN-γ responses than IL-2 [61.9% (34/55) IFN-γ vs 56.4% (31/55) IL-2; and 65.4% (36/55) had at least a positive result for one of the two cytokines (p = 0.66)]. Positive responses in the acute group tended to increase with severity for both cytokines, but severe patients under IMV had a lower number of IL-2 positive results than those under NIV [71% (22/31) for NIV vs 62.5% (5/8) for IMV]. When positivity was analyzed according to the days of hospitalization in the acute COVID-19 patients, positive responses tended to increase after hospitalization except for samples taken on day 28 (Supplementary Table 3). Quantity of IFN-γ or IL-2 response was analyzed in the 17 monitored patients, and high inter-individual variability was observed over time. No differences were observed when comparing the evolution of the response through the follow-up according to disease outcome (Supplementary Fig. 1).

Convalescent individuals had higher rates of positivity when compared to acute patients for both cytokines [82.5% (80/97) in convalescent vs 61.8% (34/55) in acute patients for IFN-γ; 86.6% (84/97) in convalescent vs 56.4% (31/55) in acute for IL-2]. High amount of positive responses for at least one of the cytokines was detected in 87/97 (89.7%) of the convalescent individuals in comparison with detected IFN-γ or IL-2 alone (p = 0.023). Positive responses for IL-2 increased with the severity of the disease and for IFN-γ they followed a similar pattern except for severe NIV patients (Table 1).

**Figure 1:**
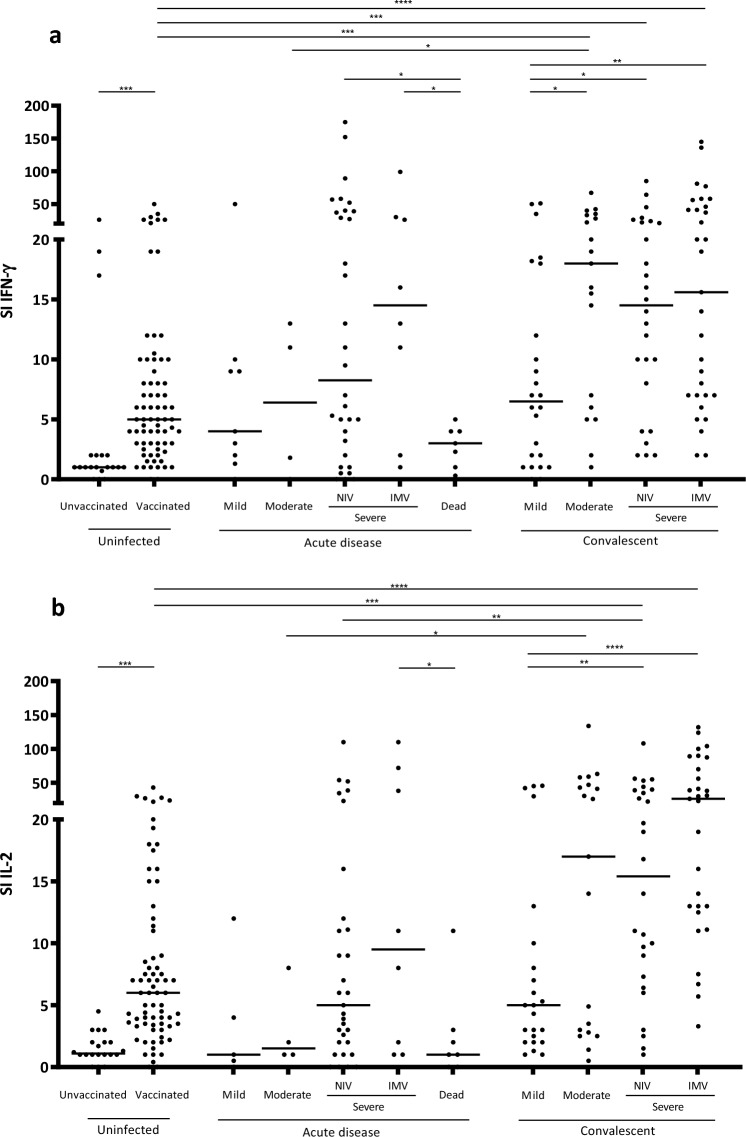
IFN-γ (**a**) and IL-2 (**b**) T-cell responses against the SARS-CoV-2 antigens. The specific response for each cytokine is represented using the SI (stimulation Index). Differences between two group conditions were calculated using the two-tailed Mann–Whitney U test. P is considered significant when < 0.05 (* < 0.05, ** < 0.01, *** < 0.001, and *** < 0.0001). SI: Stimulation index; Non-Invasive Ventilation (NIV); Invasive Mechanical Ventilation (IMV).

As shown in Fig. 1a and b, when analyzing the quantity of specific IFN-γ and IL-2 T-cell responses (SI values) in the SARS-CoV-2 panel, as expected, significantly higher response was triggered in the uninfected vaccinated individuals compared to the unvaccinated for both cytokines (p < 0.0001). On the other hand, when analyzing the quantity of specific IFN-γ and IL-2 T-cell responses (SI values), they were significantly higher in convalescent than in acute cases (p = 0.023 and p < 0.0001, respectively). Although not significant, IFN-γ and IL-2 responses in acute COVID-19 patients showed an increase with severity. Severe acute patients with IMV and NIV displayed significantly higher responses than those who did not survive the disease (for IFN-γ: p < 0.05 for both comparisons; for IL-2: p < 0.05, only significant when comparing IMV with death). In addition, the SI increased with severity in convalescent individuals. Moreover, moderate convalescent individuals showed an increased IFN-γ or IL-2 response when compared to those with moderate acute disease (p < 0.05 for both cytokines). For IL-2, differences were also significantly higher in severe patients with NIV between the convalescence and the acute phase (p < 0.01). Interestingly, IFN-γ and IL-2 responses were higher in severe convalescent cases than in vaccinated individuals (for both cytokines: p = 0.0001 in convalescent individuals with IMV vs uninfected vaccinated; and p = 0.001 in convalescent individuals with NIV vs uninfected vaccineted).

**Figure 2:**
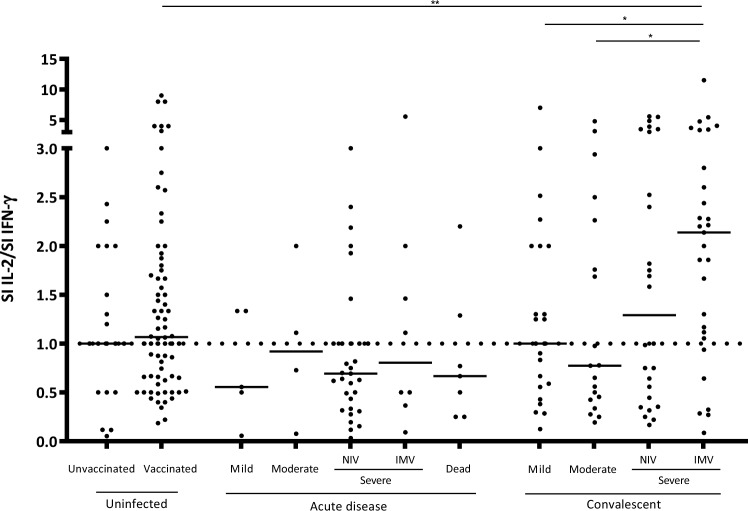
SI ratios between both cytokine responses (SI IL-2/SI IFN-γ) for the different study groups. Values over 1 meant higher IL-2 response whilst values below 1 meant higher IFN-γ response. Samples with a 0 value for IFN-γ SI and IL-2 higher than 0 were excluded for avoiding indeterminate results in the ratio calculation. Differences between conditions were calculated using the two-tailed Mann–Whitney U test. P is considered significant when < 0.05 (* < 0.05, ** < 0.01, *** < 0.001, and *** < 0.0001). SI: Stimulation index; Non-Invasive Ventilation (NIV); Invasive Mechanical Ventilation (IMV).

Considering the ratio between both responses (SI IL-2/SI IFN-γ) (Fig. 2), acute patients showed a median ratio below 1, indicating a higher quantity of IFN-γ specific responses than IL-2. In contrast, median ratios higher than 1 were obtained for vaccinated individuals and severe convalescent individuals. Although no significant differences were observed on these comparisons, we found these results relevant as there is a trend that IFN-γ is highly secreted in acute patients (ratios below 1) and IL-2 in convalescent ones (ratios for NIV and IMV above 1). To compare both responses, a correlation between the SI of both IFN-γ and IL-2 from each of the samples was performed. Globally, a high correlation was observed between both responses (SR = 0.743, p < 0.0001). This correlation was also performed in uninfected vaccinated, acute, and convalescent groups, showing a moderate correlation in vaccinated individuals (SR = 0.58, p < 0.0001), and a high correlation in acute and convalescent patients (SR = 0.805, p < 0.0001 for acute patients; SR = 0.7, p < 0.0001 for convalescent, Supplementary Fig. 2a–c, respectively).

### T-cell response with time differs according to immunization through vaccination or disease

**Figure 3:**
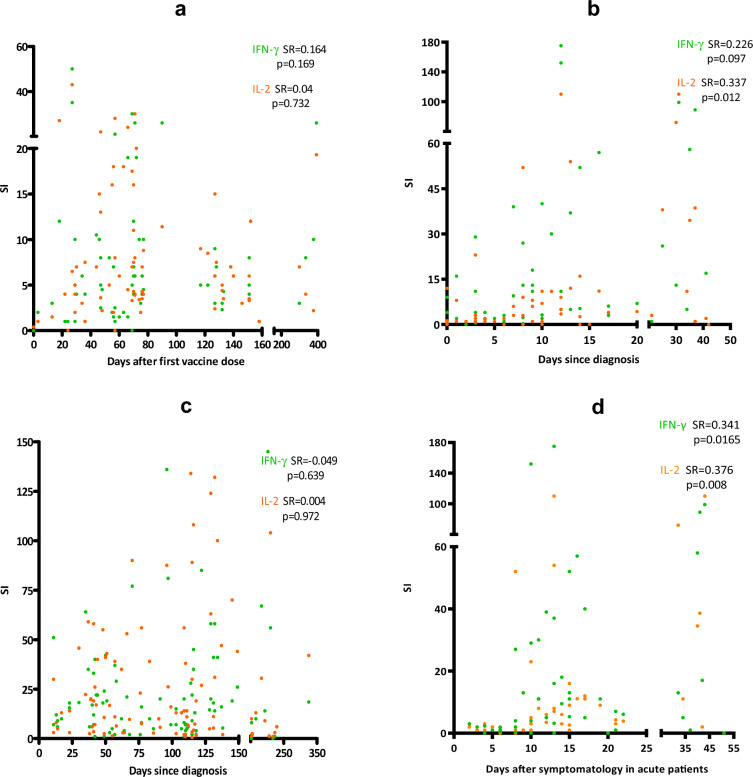
Correlations between IFN-γ (green) and IL-2 (orange) T-cell response with time since the first dose of the vaccine administration (**a**); time since diagnosis in acute patients (**b**) and convalescent individuals (**c**); and time since symptomatology appearance in acute patients (**d**). For figure (**b**), some samples were collected 35 days post-diagnosis during patient hospitalization in semi-critical or in intensive care units with severe symptomatology. For (**c**), some samples were collected 10 days after diagnosis but are considered convalescent as they correspond to healthcare workers with mild symptoms or without symptoms during the infection and have either a posterior negative PCR during their routine examinations or a finalization of the symptomatology. The specific response for each cytokine is represented using the SI. Correlations were calculated using the two-tailed non-parametric Spearman test. SI: Stimulation index.

Correlations between IFN-γ/IL-2 T-cell responses and time after vaccination were performed. No significant correlation was found between T-cell responses and time after vaccination for uninfected vaccinated individuals (SR = 0.164, p = 0.169 for IFN-γ; SR = 0.04, p = 0.732 for IL-2, Fig. 3a). In addition, after the second dose of the vaccine, 47 of the 66 samples tested (71.2%) showed an IFN-γ T-cell response, while 56 of the 66 (84.8%) showed an IL-2 T-cell one. Fifty-eight of the 66 samples with at least two vaccine doses (87.9%) had any response to IFN-γ and/or IL-2 response.

Responses for both cytokines tended to increase over time in acute patients, being significant in the case of IL-2 response (SR = 0.226, p = 0.097 for IFN-γ; SR = 0.332, p = 0.012 for IL-2, Fig. 3b). The same correlation was performed for convalescent patients and no significant increased response through time was seen for either IFN-γ or IL-2 (SR = 0.049, p = 0.64 for IFN-γ, SR = 0.004, p = 0.97 for IL-2, Fig. 3c). Regarding time since symptomatology appearance, a significant increase through time was seen for both IFN-γ and IL-2 responses (SR = 0.341, p = 0.0165 for IFN-γ; SR = 0.376, p = 0.008 for IL-2, Fig. 3d).

### T-cell and humoral immune response had moderate correlations but were impaired in some clinical situations

When analyzing humoral response excluding vaccinated uninfected individuals, IgG against nucleocapsid (NCP) was found in 133 of the 172 samples (77.3%, Table 2). Eighteen of the 55 acute patients showed IgM against NCP response (32.7%). Neutralizing antibodies were detected in 200 samples (82%), 197 of them also positive for IgGs against Spike (98.5%). IgGs against Spike had the highest rates of positive responses in the uninfected vaccinated and convalescent groups [70/72 (97.2%) in uninfected vaccinated and 96/97 (99%) in convalescent]. IgG against NCP was the humoral response most found in acute patients [45/55 (81.8%)]. Neutralizing antibodies specific for mutations in the spike antigen from other variants different to the ancestral one, as omicron variants, are detected with this assay with a low sensitivity, therefore, the percentage of neutralization can be underestimated for some participants^21^.

**Table 2:** Percentage of positive responses against SARS-CoV-2 peptides.

	IgG spike	IgG NCP	IgM NCP	Neutralizing	Any Ab
Uninfected (n = 92)	72/92 (78.3)	4/92 (4.4)	2/92 (2.2)	70/92 (76.1)	73/92 (79.3)
Unvaccinated (n = 20)	2/20 (10)	1/20 (5)	1/20 (5)	3/20 (15)	3/20 (15)
Vaccinated (n = 72)	70/72 (97.2)	3/72 (4.2)	1/72 (1.4)	67/72 (93.1)	70/72 (97.2)
Acute disease (n = 55)	38/55 (69.1)	45/55 (81.8)	18/55 (32.7)	36/55 (65.5)	48/55 (87.3)
Mild (n = 5)	3/5 (60)	3/5 (60)	1/5 (20)	3/5 (60)	3/5 (60)
Moderate (n = 4)	1/4 (25)	3/4 (75)	1/4 (25)	2/4 (50)	3/4 (75)
Severe NIV (n = 31)	25/31 (80.6)	29/31 (93.5)	11/31 (35.5)	22/31 (71)	30/31 (96.8)
Severe IMV (n = 8)	7/8 (87.5)	7/8 (87.5)	5/8 (62.5)	6/8 (75)	7/8 (87.5)
Dead (n = 7)	2/7 (28.6)	3/7 (42.9)	0/7 (0)	3/7 (42.9)	5/7 (71.4)
Convalescent (n = 97)	96/97 (99)	87/97 (89.7)	9/97 (9.3)	94/97 (96.9)	96/97 (99)
Mild (n = 23)	22/23 (95.7)	14/23 (60.9)	1/23 (4.3)	21/23 (91.4)	22/23 (95.7)
Moderate (n = 19)	19/19 (100)	18/19 (94.7)	1/19 (5.3)	18/19 (94.7)	19/19 (100)
Severe NIV (n = 26)	26/26 (100)	26/26 (100)	4/26 (15.4)	26/26 (100)	26/26 (100)
Severe IMV (n = 29)	29/29 (100)	29/29 (100)	3/29 (10.3)	29/29 (100)	29/29 (100)

**Figure 4:**
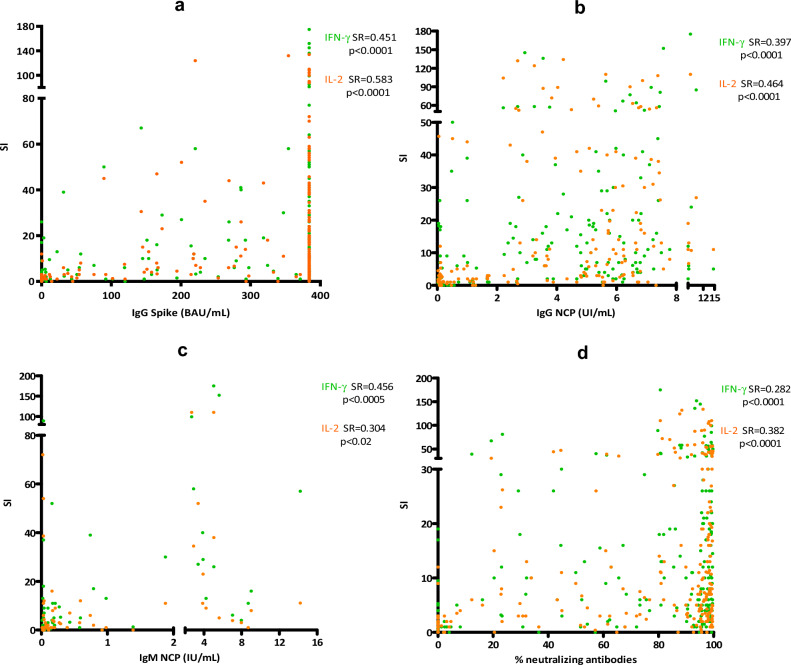
Correlations between IFN-γ (green) and IL-2 (orange) T-cell response with IgGs against Spike (**a**), IgGs against NCP (excluding the uninfected vaccinated individuals) (**b**), IgMs in acute patients (**c**), and per-centage of neutralization (**d**). The specific response for each cytokine is represented using the SI. Correlations were calculated using the two-tailed non-parametric Spearman test. In (**a**), results equal to or over 384 BAU/mL were excluded for statistical analysis (the results including the ones over > 384 BAU/ml maintained significance and moderate correlations: SR IFN-γ = 0.317; SR IL-2 = 0.429). SI: Stimulation index. BAU: Binding antibody units.

Both T-cell and humoral immune response were analyzed and correlated to detect those impaired cases and thus increase the number of specific response detections. IgG response showed significant moderate correlations with both IFN-γ (SR = 0.451, p < 0.0001 for IgGs against Spike excluding > 384 BAU/mL results, and SR = 0.397, p < 0.0001 for IgG against NCP) and IL-2 responses (SR = 0.583, p < 0.0001 for IgG against Spike excluding > 384 BAU/mL results, and SR = 0.464, p < 0.0001 for IgG against NCP; Fig. 4a and b). A moderate significant correlation was seen in acute individuals between IgM response against NCP and IFN-γ response (SR = 0.456, p = 0.0005), and a weak correlation with IL-2 response (SR = 0.304, p = 0.02, Fig. 4c). Weak correlations were found between the percentage of neutralization and IFN-γ/IL-2 T-cell responses (SR = 0.282, p < 0.0001 for IFN-γ and SR = 0.382, p < 0.0001, Fig. 4d).

Discrepancies between T-cells (positive for IFN-γ and/or IL-2) and the different humoral responses (IgG against Spike, IgG against NCP, and neutralizing antibodies) were analyzed (Supplementary Tables 4, 5 and 6). Most of the discrepancies were due to having a humoral but not a T-cell response. When comparing T-cell with Spike IgG response, 38 discrepancies were found [38/244 (15.6%)], most of them (28/38, 73.7%) due to having positive IgG response against Spike but no cellular one (neither IFN- γ nor IL-2) [in particular, 11/72 (15.3%) of these discrepancies were found in uninfected vaccinated individuals, 7/55 (12.7%) in acute patients, and 9/97 (9.3%) in convalescents; supplementary Table 6]. Similarly, positive humoral and negative T-cells responses discrepancies were found when comparing nucleocapsid IgGs [13/55 (23.6%) in acute patients, and 7/97 (7.2%) in convalescents; Supplementary Table 6] or neutralizing antibodies with cytokine responses [11/72 (15.3%) in uninfected vaccinated individuals; 15/55 (27.3%) in acute patients, and 9/97 (9.3%) in convalescents; Supplementary Table 6]. On the contrary, more positive cellular immune responses were detected when compared with IgM against nucleocapsid in acute patients. Interestingly, a total of 21 from 55 patients (38.2%) with acute disease had a positive T-cell response (IFN-γ and/or IL-2), but a negative IgM against nucleocapsid response (Supplementary Table 6). Therefore, the T-cell response still contributed by detecting some specific responses against the pathogen when the humoral response was not found, particularly in acute patients (Supplementary Tables 4, 5 and 6).

## Discussion

Understanding the T-cell response to SARS-CoV-2 is crucial to determine cellular immunity generated after infection or vaccination as well as to characterize long-term immunity and protection against reinfection. While IFN-γ has been shown as crucial, other cytokines such as IL-2 may also play an important role in memory and protection against SARS-CoV-2. In this study, we assessed IFN-γ and IL-2 released by T-cells from COVID-19 patients and from vaccinated as well as unvaccinated uninfected individuals. This response was also compared to the humoral one.

Our main findings suggest that T-cell response is lower during the acute phase of the disease in comparison to convalescence. Moreover, IL-2 cytokine was higher than IFN-γ in vaccinated and convalescent individuals. Our results also indicate that although some discrepancies in antibody-positive and T-cell-negative responses were detected, a good correlation between humoral-cellular responses was observed. In our study, an increased response of both IFN-γ and IL-2 cytokines was seen in convalescent individuals, in comparison to acute COVID-19 patients. This finding has been previously described for IFN-γ, being attributed to a presumably not well-established adaptive immune response and, as a consequence, a very low specific cytokine secretion in acute patients^11,22,23^. In addition, when comparing results between both cytokines, IFN-γ release was higher than that of IL-2 during acute disease, contrary to the convalescent phase where higher IL-2 levels were observed instead. Moreover, when combining IL-2 with IFN-γ the percentage of positive responders increased, pointing out the overlooked information when only measuring IFN-γ^15,24^. As reported in other studies, effector T-cells probably secrete IFN-γ, since it is the cellular subset actively fighting the infection, whereas IL-2 would be secreted by memory T-cells^25^. Furthermore, IL-2 can represent a good protection biomarker as it has a central role in the maintenance of memory T-cell populations and their effector functions, being secreted mainly by memory CD4+ T-cells and enhancing the activity of both NK and CD8+ T-cells^26^. In this sense, knowing the roles that both cytokines play in the adaptive response against SARS-CoV-2, would allow an understanding of immune protection against the virus and reinfection, as reported for other viruses^26^. It should also not be overlooked that innate immune response is also participating in the release of IFN-γ in a smaller level by means of NK cells found inside the PBMCs population, and that this cellular subset is fundamental in the beginning of the infection. Further studies in this way should also be performed^27^.

It is important to mention that COVID-19 severe patients with a fatal outcome (death) showed particularly diminished T-cell responses when compared to other groups. Lymphopenia and anergy due to apoptosis in some immune cellular subsets, particularly cytotoxic T-cells, have been reported in critical COVID-19 patients^23,28–30^. These patients are described as having a weakened immunity to SARS-CoV-2 due to an inability to mount a functional specific adaptive response to preclude the infection and stop viral replication, and as a result, have an unrestrained dissemination of the infection leading to death^28–31^. Moreover, it is observed here that T-cell responses increased according to the severity (excluding death) in those acute and convalescent patients, resulting in high levels of IFN-γ and even higher of IL-2 during severe disease compared with mild forms. This can be explained as a result of high viral loads in those severe patients during the acute phase^32,33^. On the contrary, it has been reported that disease severity is not related to specific CD4+ T-cells and CD8+ T-cell subsets secreting IFN-γ and IL-2, but to other inflammatory cytokines being secreted aberrantly, and being related to a worse infection and tissue damage^34^. Although our results indicate that specific IFN-γ and IL-2 cytokines are increased during severity, we hypothesize that their release is a consequence of a severe inflammatory scenario to avoid a fatal outcome, and could be used as a prognosis biomarker for clinical management of severe COVID-19 patients. Therefore, the lack of response would indicate that the patient is not producing a good inflammatory response and could die due to the inability to fight SARS-CoV-2 infection. These findings collide with our previous study^11^, as we described that severe COVID-19 patients had lower IFN-γ responses. A possible explanation for this contradiction is that in this prior study, severe patients were not divided into two groups depending on the ventilatory requirements, including also patients with fatal outcomes in the same group, and as a consequence, affecting directly the decrease of T-cell responses in severe individuals. Moreover, another immunoassay was used for the previous study and therefore different approaches in the design between the tests may be affecting to the final results.

When comparing the specific response after infection with that generated after vaccination, our data indicates that specific T-cell responses were significantly higher in convalescents than in non-infected vaccinated individuals. Controversial data in this matter has been described, as some studies report a similar response after vaccination than that found during convalescence; nevertheless, others are in line with those reported here^11,35^. In this sense, some studies have reported an increased risk of reinfection in vaccinated individuals when compared to convalescent ones or individuals with hybrid immunity^24,35,36^. In line with that, assessing the overall T-cell response with a pool of SARS-CoV-2 antigens can provide information about this increased protection against reinfection, as response against all the antigens is found when having had previous infection (convalescent individuals and individuals with hybrid immunity). Differences observed between the immune response generated after vaccination and infection reside on the type of immunity triggered. Vaccination is focused both in humoral and cellular induction. The role of humoral response is to prevent infection, but it has been shown to be waning few years after immunization; instead, memory T-cell response has an essential role in long-term immunity, as seen during convalescence, and has been seen to be more durable^37–39^. In addition, vaccine design has only been centered on triggering a limited response against Spike antigen, leaving behind nucleocapsid and membrane antigens which would give a broader protection against new infections. Moreover, T-cell response has been seen to be cross-protective to fight variants of concern (VOCs) when the humoral response is compromised^28^. As discussed before, it has been shown that IL-2 is increased during convalescence, suggesting the importance of IL-2 in memory T-cell response. Therefore, the measurement of IL-2, in combination with IFN-γ, could be considered in diagnostics and the immune status evaluation, as they may determine the response conferred after vaccination or disease, providing an accurate definition of the immune cellular status of the individual against SARS-CoV-2^26^. Thus, these measurements could be a useful tool for clinicians to better manage their patients, especially to know whether a T-cell response is present or not in vaccinated immunocompromised patients with no detectable antibody responses. Interestingly, in our study, vaccinated mild convalescent individuals showed higher percentage of positive responses for both cytokines than the unvaccinated ones [9/12 (75%) vs 6/11 (55.6%) for IFN-γ, 10/12 (83.3%) vs 6/11 (55.6%) for IL-2, respectively] and is in concordance with the bibliography^24^.

When comparing T-cell and humoral responses, correlations were found between both IFN-γ and IL-2 with IgGs against Spike and NCP, and neutralizing antibodies. Nevertheless, higher amount of positive IgGs and neutralizing antibodies were obtained when compared with T-cells. Concerning humoral responses, IgGs against Spike together with neutralizing antibodies were the ones detected the most, particularly during convalescence and after vaccination. The roles of humoral-cellular immunity on SARS-CoV-2 infection still remain controversial as some authors report T-cell responses appearing previously than neutralizing antibodies after vaccination, whereas others report the opposite^40–43^. Although humoral responses are seen in a larger number of individuals after the disease and vaccination, memory T-cell responses related to the release of IL-2, are fundamental when the humoral response wanes after some time, and when the virus escapes humoral response due to the appearance of new VOCs^44,45^. Therefore, both memory T-cell immune status and humoral response evaluation are indispensable for comprehending protection against new variants and possible reinfections. Detecting both responses might indicate increased protection^44,45^.

The study has some limitations that should be addressed. In the first place, statistical strength could be affected as mild and moderate subgroups of acute COVID-19 patients have small numbers. Notwithstanding, patient groups from our study were clinically well-characterized, making it possible to assess the immune response in each clinical status. In the second place, the most prevalent SARS-CoV-2 VOCs were not reported in our study. Although this can affect the immune response interpretation, data concerning VOCs has been registered by Spanish authorities, and therefore, variants can be traced: Wuhan (until February 2021), Alpha (until June 2021), Delta (until December 2021), and Omicron (from December 2021)^46^. Another limitation of the study is the impossibility of assessing long-term immunity and protection, as we do not have a follow-up cohort through time after infection to assess possible reinfections according to its IL-2 status. However, it is well known that IL-2 secretion is associated with the presence of a memory T-cell response, which is important in the protection against possible reinfection, and it is highly produced in convalescents and after the second dose of the vaccine. Finally, vaccinated individuals could not be followed-up, therefore, the response through time and administrated doses could not be assessed. Despite that, time since the administration of the different doses was registered for all vaccinated individuals and response through time could be evaluated.

Altogether, the measurement of IFN-γ and IL-2 cytokines can have a value for SARS-CoV-2 infection management. Our results show that IFN-γ in combination with IL-2 increases response detection in acute and convalescent individuals, having IFN-γ response a role during the acute phase of the disease, and IL-2 on long-term immunity against natural immunization or vaccination. In addition, IFN-γ detection can be a useful biomarker for monitoring severe acute patients, as our results indicate that those individuals with a poor outcome have lower levels of this cytokine. Moreover, fluorescence ELISPOT technology allows the detection of these specific immune responses against SARS-CoV-2 easily, being able to be adapted in the majority of laboratories. Finally, according to the findings observed here, T-cell responses generated in acute COVID-19 patients are lower compared to convalescence, indicating that T-cell responses need time to be generated. In some cases, the lack of cellular immunity is compensated by antibodies, confirming the role of both types of immune responses in infection and vaccination, and confirming that their dual detection can increase the number of specific response detections. All these data suggest the possible role of IFN-γ and IL-2 as effector and memory cytokines, respectively. Such dual detection is promising for assessing the post-immunization status and managing the infection, but more studies are needed in this direction evaluating other cytokines or cell markers related to diagnosis and disease outcome.

## Methods

### Study samples

Two-hundred sixty-three blood samples were drawn from 232 participants at Hospital Universitari Germans Trias i Pujol (Badalona, Spain) from July 2020 to May 2022. All the participants of the study filled out and signed a written informed consent form. The study was approved by the Ethics Committee of the Hospital Universitari Germans Trias i Pujol (PI-20-117), and the experiments were performed according to current regulations and guidelines. Clinical and demographic data from the individuals included in the study are summarized in Table 3, and data from the samples concerning time since diagnosis, time since vaccine doses and lymphopenia are included in Supplementary Table 7. Individuals were classified following WHO 2020 guidelines as follows^47^:

(i) Ninety-three uninfected healthcare workers with no previous nor present positive SARS-CoV-2 test (PCR or rapid antigen test (RAT)), and/or detectable IgM or IgG plasma antibodies against the virus. They were classified into two groups: (a) unvaccinated individuals (n = 21), and (b) vaccinated individuals (n = 72), in which 91.7% (66/72) had received two doses of the vaccine.
(ii) Sixty-six samples from 35 COVID-19 patients during the acute phase of the disease and with present positive SARS-CoV-2 test. Participants were classified according to disease severity by hospitalization and required ventilation criteria into: (a) healthcare workers with mild infection (n = 8) who had a positive SARS-CoV-2 PCR in work routine screenings and were neither hospitalized nor required ventilation support during infection; (b) moderate (n = 2) when the patient required hospitalization and ventilation with nasal prongs or Ventimask (VMK); (c) severe (n = 22) when the patient required high-flow ventilation or non-invasive ventilation (NIV) (n = 17), or invasive mechanical ventilation (IMV) (n = 5); and (d) dead (n = 3) when the patient died during infection. From acute COVID-19 group, 17 patients were followed-up and two or more samples were collected during days 0, 2, 7, 28, and/or at discharge after admission in semi-critical or intensive care units (48 samples in total).
(iii) One-hundred and four individuals after overcoming the acute phase of SARS-CoV-2 infection with a previous COVID-19 positive diagnostic. These individuals were also classified following WHO 2020 guidelines according to the severity of the previous disease as: (a) mild (n = 25); (b) moderate (n = 22); and (c) severe (n = 57), with high-flow ventilation or NIV (n = 26), or IMV (n = 31).

**Table 3:** Descriptive table from patients included in the study.

Patients variables (n = 232)	Controls (n = 93)		Acute (n = 35)					Convalescent (n = 104)			
	Unvaccinated (n = 21)	Vaccinated (n = 72)	Mild (n = 8)	Moderate (n = 2)	Severe (n = 22)		Dead (n = 3)	Mild (n = 25)	Moderate (n = 22)	Severe (n = 57)	
					NIV (n = 17)	IMV (n = 5)				NIV (n = 26)	IMV (n = 31)
Age (years ± SD)	34.6 ± 10.4	41.4 ± 13.9	34.5 ± 16.6	55.1 ± 26.7	59.6 ± 13.1	63.2 ± 11.3	79.6 ± 2.3	39.9 ± 12.9	61 ± 14.2	60.6 ± 14.1	58.6 ± 10.2
Male N (%)	3 (14.3)	18 (25)	4 (50)	2 (100)	15 (88.2)	4 (80)	2 (66.7)	5 (20)	11 (50)	14 (53.8)	22 (74.2)
Pneumonia N (%)^a^	0 (0)	0 (0)	0 (0)	2 (100)	17 (100)	5 (100)	3 (100)	0 (0)	19 (86.4)	26 (100)	31 (100)
Unilobar	0 (0)	0 (0)	0 (0)	0 (0)	0 (0)	0 (0)	0 (0)	0 (0)	5 (22.7)	0 (0)	1 (3.2)
Multilobar	0 (0)	0 (0)	0 (0)	2 (100)	17 (100)	5 (100)	3 (100)	0 (0)	14 (63.6)	26 (100)	30 (96.8)
ICU admission N (%)^a^	0 (0)	0 (0)	0 (0)	0 (0)	5 (29.4)	5 (100)	2 (66.7)	0 (0)	0 (0)	2 (7.7)	30 (96.8)
Oxygen support N (%)^a^	0 (0)	0 (0)	0 (0)	2 (100)	17 (100)	5 (100)	3 (100)	0 (0)	16 (72.7)	26 (100)	31 (100)
Nasal prongs or VMK	0 (0)	0 (0)	0 (0)	2 (100)	0 (0)	0 (0)	0 (0)	0 (0)	16 (72.7)	1 (3.8)	0 (0)
Non-invasive mechanical vent	0 (0)	0 (0)	0 (0)	0 (0)	17 (100)	0 (0)	3 (100)	0 (0)	0 (0)	26 (96.2)	0 (0)
Invasive mechanical vent	0 (0)	0 (0)	0 (0)	0 (0)	0 (0)	5 (100)	0 (0)	0 (0)	0 (0)	0 (0)	31 (100)
Vaccinated with 1st dose N (%)^b^	0 (0)	72 (100)	2 (25)	0 (0)	1 (5.9)	0 (0)	0 (0)	13 (52)	1 (4.5)	0 (0)	0 (0)
Vaccinated with 2nd dose N (%)^b^	0 (0)	66 (91.7)	0 (0)	0 (0)	0 (0)	0 (0)	0 (0)	10 (40)	0 (0)	0 (0)	0 (0)
Vaccinated with 3rd dose N (%)	0 (0)	3 (4.2)	0 (0)	0 (0)	0 (0)	0 (0)	0 (0)	2 (8)	0 (0)	0 (0)	0 (0)
Comorbidities N (%)	1 (4.7)	8 (11.1)	0 (0)	1 (50)	15 (88.2)	3 (60)	3 (100)	3 (12)	11 (50)	21 (80.8)	21 (67.7)
Respiratory disorders (asthma, OSAS, COPD)	0 (0)	2 (2.8)	0 (0)	1 (50)	6 (35.3)	0 (0)	0 (0)	0 (0)	8 (36.4)	3 (11.5)	3 (9.7)
Cardiovascular diseases (AHT, ictus, atrial fibrillation)	1 (4.7)	2 (2.8)	0 (0)	1 (50)	7 (41.2)	3 (60)	3 (100)	1 (4)	6 (27.3)	14 (53.8)	15 (48.4)
Autoimmune disorders (DM2, psoriasis, Jorgen, other)	0 (0)	3 (4.2)	0 (0)	0 (0)	6 (35.3)	1 (20)	1 (33.3)	2 (8)	1 (4.5)	6 (23.1)	6 (19.4)
Central nervous system disorders (dementia, epilepsy, Parkinson)	0 (0)	1 (1.4)	0 (0)	0 (0)	2 (11.8)	0 (0)	1 (33.3)	0 (0)	1 (4.5)	0 (0)	1 (3.2)
Malignant neoplasies	0 (0)	0 (0)	0 (0)	0 (0)	0 (0)	1 (20)	1 (33.3)	0 (0)	1 (4.5)	3 (11.5)	2 (6.4)
Obesity	0 (0)	0 (0)	0 (0)	0 (0)	7 (41.2)	1 (20)	0 (0)	0 (0)	3 (13.6)	4 (15.3)	7 (22.6)
Immunosuppressive treatment N (%)	0 (0)	4 (5.6)	1 (12.5)	0 (0)	4 (23.5)	0 (0)	1 (33.3)	1 (4)	4 (18.2)	3 (11.5)	2 (6.4)
Oral (prednisone, NSAIDS, etc.)	0 (0)	1 (1.4)	1 (12.5)	0 (0)	2 (11.8)	0 (0)	1 (33.3)	0 (0)	4 (18.2)	0 (0)	1 (3.2)
Inhaled	0 (0)	2 (2.8)	0 (0)	0 (0)	2 (11.8)	0 (0)	0 (0)	0 (0)	0 (0)	2 (7,7)	1 (3.2)
Topic	0 (0)	2 (2.8)	0 (0)	0 (0)	0 (0)	0 (0)	0 (0)	1 (4)	0 (0)	1 (3.8)	0 (0)
Deaths N (%)	0 (0)	0 (0)	0 (0)	0 (0)	0 (0)	0 (0)	3 (100)	0 (0)	0 (0)	0 (0)	0 (0)

*NIV* non-invasive ventilation, *IMV* invasive mechanical ventilation, *n/a* not available.

^a^In the convalescent group, these variables refer to the characteristics of their acute COVID-19 episode.

^b^Sixty-six of 72 uninfected vaccinated individuals (91.7%) with two Pfizer doses.

### Isolation of peripheral blood mononuclear cells (PBMCs)

Sixteen milliliters of blood were collected in cell preparation tubes (CPT; Becton Dickinson Diagnostics, Franklin Lakes, NJ) for density gradient PBMCs isolation. After centrifugation, PBMCs were collected, washed with 10% FBS RPMI (Biowest, Nuaillé, France), and counted using trypan blue. Cells were cryopreserved in FBS 10% DMSO (Sigma-Aldrich, Saint Louis, United States of America), first at − 80 °C in a Nalgene Mr. Frosty Cryo 1 °C Freezing Container (ThermoFisher, Waltham, United States of America) and were then transferred to liquid nitrogen within a week.

### Detection of IFN-γ and IL-2 T-cell responses using a fluorescence ELISPOT assay

Fluorescent ELISPOT is a technique that allows the multiple detection of cytokines release against a pathogen with a very accessible protocol and without demanding a complicated analysis when compared to alternative ways to assess cellular immunity. It can also provide a result within 1 or 2 days and it can be easily implemented (not demanding expensive infrastructure and highly trained personnel). In this study, IFN-γ and IL-2 T-cell responses were measured with a fluorescence ELISPOT assay (CoV-iSpot, Autoimmun Diagnostika GmbH, Straßberg. Germany). Two-hundred thousand cells were dispensed in each well/condition in a 96-well plate coated with antibodies specific for IFN-γ and IL-2. Cells were stimulated overnight at 37 °C with two antigen pools: (a) pancoronavirus peptide mix, based on homology regions of the coronavirus viral family, and (b) SARS-CoV-2 peptide mix, based on peptides from Spike, NCP, membrane and envelope proteins unique for the Wuhan strain of the virus which can cross-react with new Spike mutations from the different variants. Negative (AIM-V) and positive controls (pokeweed-mitogen) were included for each sample. Samples were tested in duplicates for all conditions, strictly following manufacturer’s instructions. Fluorescent spots [spot forming cells (SFCs)] were counted with an automated plate reader (Autoimmun Diagnostika GmbH, Straßberg. Germany). Specific response against pancoronavirus and SARS-CoV-2 antigens was analyzed by performing a ratio between the average of SFCs for the specific panel and the average of SFCs in the negative control (stimulation index, SI). Results were interpreted as follows: when the negative control had less than 2 SFCs, a SI < 5 was considered a negative response, between 5 and 7 borderline, and ≥ 7 positive. On the contrary, if the negative control had 2 or more SFCs, a SI < 2 was considered negative, between 2 and 3 borderline, and ≥ 3 positive. Samples with less than 50 IFN-γ or IL-2 spots in the positive control or with more than 10 IFN-γ or 20 IL-2 spots in the negative control were considered indeterminate, unless positive control was invalid and response against one of the antigen pools was found. Ratios between IL-2 and IFN-γ T-cell responses were also performed (SI IL-2/SI IFN-γ).

### Detection of humoral responses with ELISA

Levels of IgGs against Spike and NCP proteins, IgM against NCP, and neutralizing antibodies were analysed by ELISA (Euroimmun, Lübek, Germany). IgGs against Spike were quantified with the QuantiVac kit; IgGs and IgMs against NCP were semi-quantified with the Anti-SARS-CoV-2 (IgG or IgM) kits; and neutralizing antibodies were measured with the NeutraLISA kit.

Plasma was incubated in a 96-well plate with specific fixed SARS-CoV-2 antigens for the measurement of IgGs against Spike, and IgG and IgM against NCP. For measuring neutralizing antibodies, plasma was incubated with the ACE2 human receptor which acts as a competitor to bind the S1/RBD domain from the SARS-CoV-2 Wuhan strain, fixed in the well.

Samples were tested in batches, having each batch a positive and a negative control to validate the test. For the semi-quantification of IgGs and IgMs against NCP, a calibrator was included to perform an absorbance ratio (AR = absorbance of the sample/absorbance of the calibrator). To quantify levels of IgG against Spike, a six-point calibration curve was used to quantify anti-Spike IgG levels [concentration given in Binding Antibody Units (BAU)/mL]. Finally, for the neutralizing antibodies ELISA, two replicas of a blank were included to calculate the percentage of neutralization [% of neutralization = 100 − ((absorbance of the sample*100)/absorbance of the blank)]. Positive, borderline, and negative cut-off values for each test were provided by the manufacturer’s instructions.

### Statistical analysis

The statistical analysis used to compare T-cell responses and ratios among groups was the two-tailed Mann–Whitney U-test for unpaired comparisons. Comparisons among the number of positives obtained in acute and convalescent cases in detecting IFN-γ, IL-2 response or response for at least one of the cytokines was calculated by a Cochran test. Statistical significance was considered when a p-value < 0.05 was obtained. Correlations were assessed by two-tailed non-parametric Spearman test. Both statistical analysis and graphical representations were done with GraphPad v8 (GraphPad Software, Inc, San Diego, CA).

### Supplementary Information

Supplementary Information.

## Data Availability

Without any reservation, raw data supporting the findings of this study will be made available by the corresponding author.
