# Supplementary material for: Measurement of IFN-γ and IL-2 for the assessment of the cellular immunity against SARS-CoV-2
Source: Sci Rep. 2024 Jan 11;14:1137. doi: 10.1038/s41598-024-51505-w (PMC10784529; doi:10.1038/s41598-024-51505-w)
Supplement: Supplementary file 1 — Supplementary Information. [file 41598_2024_51505_MOESM1_ESM.docx]

Supplementary Material

**Measurement of IFN-γ and IL-2 in the assessment of the cellular immunity against SARS-CoV-2**

**Guillem Safont^1,2,3^, Raquel Villar-Hernández^1,4^, Daria Smalchuk^1,5^, Zoran Stojanovic^2,3,6^, Alicia Marín^2,3,6^, Alicia Lacoma^1,2,3^, Cristina Pérez-Cano^7^, Anabel López-Martínez^7^, Bárbara Molina-Moya^1,2,3^, Alan Jhunior Solis^2,3,6^, Fernando Arméstar^8^, Joan Matllo^7^, Sergio Díaz-Fernández^1,2,3^, Iris Romero^1,2,3^, Irma Casas^3,9^, Kevin Strecker^4^, Rosemarie Preyer^4^, Antoni Rosell^2,3,6†^, Irene Latorre^1,2,3†^, Jose Domínguez^1,2,3†^***

*jadominguez@igtp.cat

**Supplementary Table 1.** Percentages of positivity for IFN-γ and IL-2 T-cell responses against pancoronavirus antigens

|  | **IFN-γ** | | **IL-2** | |
| --- | --- | --- | --- | --- |
|  | **PAN** | **SARS+PAN** | **PAN** | **SARS+PAN** |
| **Uninfected (n=92)** | 34/92 (37) | 27/92 (29.3) | 21/92 (22.8) | 19/92 (20.7) |
| Unvaccinated (n=20) | 1/20 (5) | 0/20 (0) | 1/20 (5) | 1/20 (5) |
| Vaccinated (n=72) | 33/72 (45.8) | 27/72 (38) | 20/72 (27.8) | 18/72 (25) |
| **Acute disease (n=55)** | 20/55 (36.4) | 18/55 (32.7) | 10/55 (18.2) | 10/55 (18.2) |
| Mild (n=5) | 0/5 (0) | 0/5 (0) | 0/5 (0) | 0/5 (0) |
| Moderate (n=4) | 0/4 (0) | 0/4 (0) | 0/4 (0) | 0/4 (0) |
| Severe NIV (n=31) | 13/31 (41.9) | 13/31 (41.9) | 7/31 (22.6) | 7/31 (22.6) |
| Severe IMV (n=8) | 4/8 (50) | 4/8 (50) | 3/8 (37.5) | 3/8 (37.5) |
| Dead (n=7) | 3/7 (42.9) | 1/7 (14.3) | 0/7 (0) | 0/7 (0) |
| **Convalescent (n=97)** | 31/97 (32) | 31/97 (32) | 39/97 (40.2) | 38/97 (39.2) |
| Mild (n=23) | 7/23 (30.4) | 7/23 (30.4) | 5/23 (21.7) | 5/23 (21.7) |
| Moderate (n=19) | 8/19 (42.1) | 8/19 (42.1) | 9/19 () | 9/19 (47.4) |
| Severe NIV (n=26) | 6/26 (23.1) | 6/26 (23.1) | 12/26 (46.2) | 11/26 (42.3) |
| Severe IMV (n=29) | 10/29 (34.5) | 10/29 (34.5) | 13/29 (44.8) | 13/29 (44.8) |

**Supplementary Table 2.** Percentages of positivity for IFN-γ+ IL-2- and IFN-γ- IL-2+ T-cell responses

|  | **IFN-γ +/ IL-2 -** | **IFN-γ -/ IL-2 +** |
| --- | --- | --- |
| **Uninfected (n=92)** | 5/92 (5.4) | 12/92 (13) |
| Unvaccinated (n=20) | 3/20 (15) | 1/20 (5) |
| Vaccinated (n=72) | 2/72 (2.8) | 11/72 (15.3) |
| **Acute disease (n=55)** | 5/55 (9.1) | 2/55 (3.6) |
| Mild (n=5) | 1/5 (20) | 0/5 (0) |
| Moderate (n=4) | 1/4 (25) | 1/4 (25) |
| Severe NIV (n=31) | 1/31 (3.2) | 1/31 (3.2) |
| Severe IMV (n=8) | 1/8 (12.5) | 0/8 (0) |
| Dead (n=7) | 1/7 (14.3) | 0/7 (0) |
| **Convalescent (n=97)** | 3/97 (3.1) | 7/97 (7.2) |
| Mild (n=23) | 1/23 (4.3) | 2/23 (8.7) |
| Moderate (n=19) | 2/19 (10.5) | 1/19 (5.3) |
| Severe NIV (n=26) | 0/26 (0) | 2/26 (7.7) |
| Severe IMV (n=29) | 0/29 (0) | 2/29 (6.9) |

**Supplementary Table 3.** Percentages of positivity for acute follow-up samples

| **Days after Respiratory semi intensive care admission** | **IFN-γ** | **IL-2** | **IFN-γ+IL-2** | **IgG Spike** | **IgG NCP** | **IgM NCP** |
| --- | --- | --- | --- | --- | --- | --- |
| **Overall (n=50)** | 32 (64) | 30 (60) | 34 (68) | 35 (70) | 42 (84) | 17 (34) |
| **Day 0 (n=12)** | 4 (33.3) | 3 (25) | 4 (33.3) | 3 (25) | 8 (66.7) | 4 (33.3) |
| **Day 2 (n=16)** | 10 (62.5) | 8 (50) | 11 (68.8) | 11 (68.8) | 13 (80.3) | 6 (37.5) |
| **Day 7 (n=9)** | 7 (77.7) | 8 (88.8) | 8 (88.8) | 9 (100) | 9 (100) | 2 (22.2) |
| **Discharge (n=5)** | 5 (100) | 5 (100) | 5 (100) | 4 (80) | 4 (80) | 3 (60) |
| **Day 28 (n=8)** | 6 (75) | 6 (75) | 6 (75) | 8 (100) | 8 (100) | 2 (25) |

**Supplementary Table 4.** IFN-γ results compared to the rest of the results obtained.

| **IFN-γ** | | IL-2 | | IgG Spike | | IgG NCP | | IgM NCP | | Neutralizing | |
| --- | --- | --- | --- | --- | --- | --- | --- | --- | --- | --- | --- |
| Uninfected (n=92) | | Pos | Neg | Pos | Neg | Pos | Neg | Pos | Neg | Pos | Neg |
| IFN-γ | Pos | 47 | 5 | 48 | 4 | 3 | 49 | 0 | 52 | 48 | 4 |
|  | Neg | 12 | 28 | 24 | 16 | 1 | 39 | 2 | 38 | 22 | 18 |
| *Unvaccinated (n=20)* | |  |  |  |  |  |  |  |  |  |  |
| IFN-γ | Pos | 0 | 3 | 0 | 3 | 0 | 3 | 0 | 3 | 1 | 2 |
|  | Neg | 1 | 16 | 2 | 15 | 1 | 16 | 1 | 16 | 2 | 15 |
| *Vaccinated (n=72)* | |  |  |  |  |  |  |  |  |  |  |
| IFN-γ | Pos | 47 | 2 | 48 | 1 | 3 | 46 | 0 | 49 | 47 | 2 |
|  | Neg | 11 | 12 | 22 | 1 | 0 | 23 | 1 | 22 | 20 | 3 |
| Acute patients (n=55) | |  |  |  |  |  |  |  |  |  |  |
| IFN-γ | Pos | 29 | 5 | 29 | 5 | 30 | 4 | 15 | 19 | 28 | 6 |
|  | Neg | 2 | 18 | 8 | 12 | 15 | 5 | 2 | 18 | 8 | 13 |
| *Mild (n=5)* | |  |  |  |  |  |  |  |  |  |  |
| IFN-γ | Pos | 1 | 1 | 0 | 2 | 0 | 2 | 0 | 2 | 0 | 2 |
|  | Neg | 0 | 3 | 3 | 0 | 3 | 0 | 1 | 2 | 3 | 0 |
| *Moderate (n=4)* | |  |  |  |  |  |  |  |  |  |  |
| IFN-γ | Pos | 1 | 1 | 1 | 1 | 2 | 0 | 1 | 1 | 2 | 0 |
|  | Neg | 1 | 1 | 0 | 2 | 1 | 1 | 0 | 2 | 0 | 2 |
| *Severe NIV (n=31)* | |  |  |  |  |  |  |  |  |  |  |
| IFN-γ | Pos | 21 | 1 | 20 | 2 | 21 | 1 | 9 | 13 | 18 | 4 |
|  | Neg | 1 | 8 | 5 | 4 | 9 | 0 | 1 | 7 | 4 | 5 |
| *Severe IMV (n=8)* | |  |  |  |  |  |  |  |  |  |  |
| IFN-γ | Pos | 5 | 1 | 6 | 0 | 6 | 0 | 5 | 1 | 6 | 0 |
|  | Neg | 0 | 2 | 1 | 1 | 1 | 1 | 0 | 2 | 0 | 2 |
| *Dead (n=7)* | |  |  |  |  |  |  |  |  |  |  |
| IFN-γ | Pos | 1 | 1 | 2 | 0 | 1 | 1 | 0 | 2 | 2 | 0 |
|  | Neg | 0 | 5 | 0 | 5 | 2 | 3 | 0 | 5 | 1 | 4 |
| Convalescent (n=97) | |  |  |  |  |  |  |  |  |  |  |
| IFN-γ | Pos | 77 | 3 | 80 | 0 | 74 | 7 | 7 | 83 | 78 | 2 |
|  | Neg | 7 | 10 | 16 | 1 | 13 | 4 | 2 | 15 | 16 | 1 |
| *Mild (n=23)* | |  |  |  |  |  |  |  |  |  |  |
| IFN-γ | Pos | 14 | 1 | 15 | 0 | 10 | 5 | 0 | 15 | 14 | 1 |
|  | Neg | 2 | 6 | 7 | 1 | 3 | 5 | 1 | 7 | 7 | 1 |
| *Moderate (n=19)* | |  |  |  |  |  |  |  |  |  |  |
| IFN-γ | Pos | 15 | 2 | 17 | 0 | 16 | 1 | 1 | 16 | 16 | 1 |
|  | Neg | 1 | 1 | 2 | 0 | 2 | 0 | 0 | 2 | 2 | 0 |
| *Severe NIV (n=26)* | |  |  |  |  |  |  |  |  |  |  |
| IFN-γ | Pos | 21 | 0 | 21 | 0 | 21 | 0 | 3 | 18 | 21 | 0 |
|  | Neg | 2 | 3 | 5 | 0 | 5 | 0 | 1 | 4 | 5 | 0 |
| *Severe IMV (n=29)* | |  |  |  |  |  |  |  |  |  |  |
| IFN-γ | Pos | 27 | 0 | 27 | 0 | 27 | 0 | 3 | 24 | 27 | 0 |
|  | Neg | 2 | 0 | 2 | 0 | 2 | 0 | 0 | 2 | 2 | 0 |

**Supplementary Table 5.** IL-2 results compared to the rest of the results obtained.

| **IL-2** | | IFN-g | | IgG Spike | | IgG NCP | | IgM NCP | | Neutralizing | |
| --- | --- | --- | --- | --- | --- | --- | --- | --- | --- | --- | --- |
| Uninfected (n=92) | | Pos | Neg | Pos | Neg | Pos | Neg | Pos | Neg | Pos | Neg |
| IL-2 | Pos | 47 | 12 | 58 | 1 | 3 | 56 | 1 | 58 | 57 | 2 |
|  | Neg | 5 | 28 | 14 | 19 | 1 | 32 | 1 | 32 | 13 | 20 |
| *Unvaccinated (n=20)* | |  |  |  |  |  |  |  |  |  |  |
| IL-2 | Pos | 0 | 1 | 1 | 0 | 0 | 1 | 0 | 1 | 1 | 0 |
|  | Neg | 3 | 16 | 1 | 18 | 1 | 18 | 1 | 18 | 2 | 17 |
| *Vaccinated (n=72)* | |  |  |  |  |  |  |  |  |  |  |
| IL-2 | Pos | 47 | 11 | 57 | 1 | 3 | 55 | 1 | 57 | 56 | 2 |
|  | Neg | 2 | 12 | 13 | 1 | 0 | 14 | 0 | 14 | 11 | 3 |
| Acute patients (n=55) | |  |  |  |  |  |  |  |  |  |  |
| IL-2 | Pos | 29 | 2 | 28 | 3 | 29 | 2 | 13 | 18 | 26 | 5 |
|  | Neg | 5 | 18 | 9 | 14 | 16 | 7 | 4 | 19 | 10 | 14 |
| *Mild (n=5)* | |  |  |  |  |  |  |  |  |  |  |
| IL-2 | Pos | 1 | 0 | 0 | 1 | 0 | 1 | 0 | 1 | 0 | 1 |
|  | Neg | 1 | 3 | 3 | 1 | 3 | 1 | 1 | 3 | 3 | 1 |
| *Moderate (n=4)* | |  |  |  |  |  |  |  |  |  |  |
| IL-2 | Pos | 1 | 1 | 1 | 1 | 2 | 0 | 0 | 2 | 1 | 1 |
|  | Neg | 1 | 1 | 0 | 2 | 1 | 1 | 1 | 1 | 1 | 1 |
| *Severe NIV (n=31)* | |  |  |  |  |  |  |  |  |  |  |
| IL-2 | Pos | 21 | 1 | 21 | 1 | 21 | 1 | 9 | 13 | 19 | 3 |
|  | Neg | 1 | 8 | 4 | 5 | 9 | 0 | 1 | 7 | 3 | 6 |
| *Severe IMV (n=8)* | |  |  |  |  |  |  |  |  |  |  |
| IL-2 | Pos | 5 | 0 | 5 | 0 | 5 | 0 | 4 | 1 | 5 | 0 |
|  | Neg | 1 | 2 | 2 | 1 | 2 | 1 | 1 | 2 | 1 | 2 |
| *Dead (n=7)* | |  |  |  |  |  |  |  |  |  |  |
| IL-2 | Pos | 1 | 0 | 1 | 0 | 1 | 0 | 0 | 1 | 1 | 0 |
|  | Neg | 1 | 5 | 1 | 5 | 2 | 4 | 0 | 6 | 2 | 4 |
| Convalescent (n=97) | |  |  |  |  |  |  |  |  |  |  |
| IL-2 | Pos | 77 | 7 | 84 | 0 | 77 | 7 | 3 | 81 | 82 | 2 |
|  | Neg | 3 | 10 | 12 | 1 | 10 | 3 | 1 | 12 | 12 | 1 |
| *Mild (n=23)* | |  |  |  |  |  |  |  |  |  |  |
| IL-2 | Pos | 14 | 2 | 16 | 0 | 10 | 6 | 0 | 16 | 15 | 1 |
|  | Neg | 1 | 6 | 6 | 1 | 4 | 3 | 0 | 7 | 6 | 1 |
| *Moderate (n=19)* | |  |  |  |  |  |  |  |  |  |  |
| IL-2 | Pos | 15 | 1 | 16 | 0 | 15 | 1 | 0 | 16 | 15 | 1 |
|  | Neg | 2 | 1 | 3 | 0 | 3 | 0 | 1 | 2 | 3 | 0 |
| *Severe NIV (n=26)* | |  |  |  |  |  |  |  |  |  |  |
| IL-2 | Pos | 21 | 2 | 23 | 0 | 23 | 0 | 1 | 22 | 23 | 0 |
|  | Neg | 0 | 3 | 3 | 0 | 3 | 0 | 0 | 3 | 3 | 0 |
| *Severe IMV (n=29)* | |  |  |  |  |  |  |  |  |  |  |
| IL-2 | Pos | 27 | 2 | 29 | 0 | 29 | 0 | 2 | 27 | 29 | 0 |
|  | Neg | 0 | 0 | 0 | 0 | 0 | 0 | 0 | 0 | 0 | 0 |

**Supplementary Table 6.** IFN-γ and/or IL-2 results compared to the rest of the results obtained.

| **IFN-γ and/or IL-2** | | IgG NCP | | IgG Spike | | IgM NCP | | Neutralizing | | Ab | |
| --- | --- | --- | --- | --- | --- | --- | --- | --- | --- | --- | --- |
| Uninfected (n=92) | | Pos | Neg | Pos | Neg | Pos | Neg | Pos | Neg | Pos | Neg |
| IFN-γ + IL-2 | Pos | 3 | 61 | 60 | 4 | 1 | 63 | 60 | 4 | 61 | 3 |
|  | Neg | 1 | 27 | 12 | 16 | 1 | 27 | 10 | 18 | 13 | 15 |
| *Unvaccinated (n=20)* | |  |  |  |  |  |  |  |  |  |  |
| IFN-γ + IL-2 | Pos | 0 | 4 | 1 | 3 | 0 | 4 | 2 | 2 | 2 | 2 |
|  | Neg | 1 | 15 | 1 | 15 | 1 | 15 | 1 | 15 | 2 | 14 |
| *Vaccinated (n=72)* | |  |  |  |  |  |  |  |  |  |  |
| IFN-γ + IL-2 | Pos | 3 | 57 | 59 | 1 | 1 | 59 | 58 | 2 | 59 | 1 |
|  | Neg | 0 | 12 | 11 | 1 | 0 | 12 | 9 | 3 | 11 | 1 |
| Acute patients (n=55) | |  |  |  |  |  |  |  |  |  |  |
| IFN-γ + IL-2 | Pos | 32 | 4 | 30 | 6 | 15 | 21 | 29 | 7 | 33 | 3 |
|  | Neg | 13 | 5 | 7 | 11 | 3 | 16 | 7 | 12 | 15 | 4 |
| *Mild (n=5)* | |  |  |  |  |  |  |  |  |  |  |
| IFN-γ + IL-2 | Pos | 0 | 2 | 0 | 2 | 0 | 2 | 0 | 2 | 0 | 2 |
|  | Neg | 3 | 0 | 3 | 0 | 1 | 2 | 3 | 0 | 3 | 0 |
| *Moderate (n=4)* | |  |  |  |  |  |  |  |  |  |  |
| IFN-γ + IL-2 | Pos | 3 | 0 | 1 | 2 | 1 | 2 | 2 | 1 | 3 | 0 |
|  | Neg | 0 | 1 | 0 | 1 | 0 | 1 | 0 | 1 | 0 | 1 |
| *Severe NIV (n=31)* | |  |  |  |  |  |  |  |  |  |  |
| IFN-γ + IL-2 | Pos | 22 | 1 | 21 | 2 | 9 | 14 | 19 | 4 | 22 | 1 |
|  | Neg | 7 | 1 | 4 | 4 | 2 | 6 | 3 | 5 | 8 | 0 |
| *Severe IMV (n=8)* | |  |  |  |  |  |  |  |  |  |  |
| IFN-γ + IL-2 | Pos | 6 | 0 | 6 | 0 | 5 | 1 | 6 | 0 | 6 | 0 |
|  | Neg | 1 | 1 | 1 | 1 | 0 | 2 | 0 | 2 | 1 | 1 |
| *Dead (n=7)* | |  |  |  |  |  |  |  |  |  |  |
| IFN-γ + IL-2 | Pos | 1 | 1 | 2 | 0 | 0 | 2 | 2 | 0 | 2 | 0 |
|  | Neg | 2 | 3 | 0 | 5 | 0 | 5 | 1 | 4 | 3 | 2 |
| Convalescent (n=97) | |  |  |  |  |  |  |  |  |  |  |
| IFN-γ + IL-2 | Pos | 80 | 7 | 87 | 0 | 4 | 83 | 85 | 2 | 87 | 0 |
|  | Neg | 7 | 3 | 9 | 1 | 0 | 10 | 9 | 1 | 9 | 1 |
| *Mild (n=23)* | |  |  |  |  |  |  |  |  |  |  |
| IFN-γ + IL-2 | Pos | 11 | 6 | 17 | 0 | 0 | 17 | 16 | 1 | 17 | 0 |
|  | Neg | 3 | 3 | 5 | 1 | 0 | 6 | 5 | 1 | 5 | 1 |
| *Moderate (n=19)* | |  |  |  |  |  |  |  |  |  |  |
| IFN-γ + IL-2 | Pos | 17 | 1 | 18 | 0 | 2 | 16 | 17 | 1 | 18 | 0 |
|  | Neg | 1 | 0 | 1 | 0 | 0 | 1 | 1 | 0 | 1 | 0 |
| *Severe NIV (n=26)* | |  |  |  |  |  |  |  |  |  |  |
| IFN-γ + IL-2 | Pos | 23 | 0 | 23 | 0 | 2 | 21 | 23 | 0 | 23 | 0 |
|  | Neg | 3 | 0 | 3 | 0 | 0 | 3 | 3 | 0 | 3 | 0 |
| *Severe IMV (n=29)* | |  |  |  |  |  |  |  |  |  |  |
| IFN-γ + IL-2 | Pos | 29 | 0 | 29 | 0 | 2 | 27 | 29 | 0 | 29 | 0 |
|  | Neg | 0 | 0 | 0 | 0 | 0 | 0 | 0 | 0 | 0 | 0 |

**Supplementary Table 7.** Descriptive table of samples included in the study

| **SAMPLES VARIABLES**  **(n=263)** | | **Controls (n=93)** | | **Acute (n=66)** | | | | | **Convalescent (n=104)** | | | |
| --- | --- | --- | --- | --- | --- | --- | --- | --- | --- | --- | --- | --- |
|  |  | Unvaccinated (n=21) | Vaccinated (n=72) | Mild (n=8) | Moderate (n=4) | Severe (n=47) | | Dead (n=7) | Mild (n=25) | Moderate (n=22) | Severe (n=57) | |
|  |  |  |  |  |  | NIV (n=37) | IMV (n=10) |  |  |  | NIV (n=26) | IMV (n=31) |
| Time since diagnosis (days± SD) | | - | - | 6.1±6.9 | 6±3.6 | 16.4±12.2 | 12.8±11.9 | 9.4±11.2 | 107.8±88.3 | 72.5±46.3 | 81.2±39.3 | 116.4±33.1 |
| Time since 1^st^ vaccine dose | | - | 89±76.3 | 9.5±0 | - | 20±0 | - | - | 208.4±148.5 | 85±0 | - | - |
| Time since 2^nd^ vaccine dose | | - | 73.2±75.8 | - | - | - | - | - | 221.3±149.6 | 57±0 | - | - |
| Time since 3^rd^ vaccine dose | | - | 84±59.8 | - | - | - | - | - | 156.5±4.9 | - | - | - |
| Lymphopenia at  sampling N (%)* | *Yes* | 0 (0) | 0 (0) | 0 (0) | 2 (50) | 10 (27) | 5 (50) | 6 (85.8) | 0 (0) | 1 (4.5) | 4 (15.4) | 0 (0) |
|  | *No* | 6 (28.6) | 3 (4.2) | 0 (0) | 0 (0) | 20 (54.1) | 2 (20) | 1 (14.2) | 1 (4) | 19 (86.4) | 22 (84.6) | 31 (100) |
|  | *Not reported* | 15 (71.4) | 69 (95.8) | 8 (100) | 2 (50) | 7 (18.9) | 3 (30) | 0 (0) | 24 (96) | 2 (9.1) | 0 (0) | 0 (0) |

*Having a concentration of lymphocytes below 1200 cells/uL was considered as lymphopenia.

 **a** **b**

**Supplementary Figure 1.** IFN-γ (A; green dots) and IL-2 (B; orange dots) T-cell response in 17 acute patients who were followed-up. Red lines indicate patients who died during the SARS-CoV-2 acute phase. The specific response for each cytokine is represented using the SI

 **a** **b** **c**

**Supplementary Figure 2.** Correlations between IFN-g and IL-2 SI for, (A) uninfected vaccinated individuals, (B) acute patients, and (C) convalescent individuals. Correlations were calculated using the two-tailed non-parametric Spearman test.
